# Causes and Seasonality of Upper Respiratory Infections in Adults in Lesotho (2021–2022) (CORIAL)

**DOI:** 10.1155/cjid/3732614

**Published:** 2026-02-19

**Authors:** Nikita Sass, Linda Janita Wüthrich, Flor Lucia Gonzalez Fernandez, Emmanuel Firima, Tracy R. Glass, Moniek Bresser, Josephine Muhairwe, Bulemba Katende, Mamello Molatelle, Irene Ayakaka, Daniel Goldenberger, Kinga Feuz, Pascal Schlaepfer, Klaus Reither, Niklaus D. Labhardt

**Affiliations:** ^1^ Division Clinical Epidemiology, Department of Clinical Research, University Hospital Basel, Basel, Switzerland, unispital-basel.ch; ^2^ Division of Clinical Medicine, University of Sheffield, Sheffield, UK, sheffield.ac.uk; ^3^ Department of Medicine, Swiss Tropical and Public Health Institute, Allschwil, Switzerland, swisstph.ch; ^4^ Lesotho Country Office, SolidarMed Partnerships for Health, Maseru, Lesotho; ^5^ Research Institute of the McGill University Health Centre, Montreal, Canada, mcgill.ca; ^6^ Clinical Bacteriology & Mycology, Department of Laboratory Medicine, University Hospital Basel, Basel, Switzerland, unispital-basel.ch; ^7^ Bioinformatics, Department of Laboratory Medicine, University Hospital Basel, Basel, Switzerland, unispital-basel.ch

## Abstract

**Background:**

Upper respiratory tract infections cause morbidity and a high burden on healthcare systems worldwide, especially in low‐ and lower middle‐income countries. Recent studies throughout Africa indicate seasonal patterns that deviate from those previously described in settings with temperate climates. Currently, there are no data available on pathogens causing upper respiratory infections and their seasonal patterns in Lesotho, Southern Africa.

**Methods:**

This cross‐sectional nested study enrolled a randomly selected sample of adults presenting at St. Charles Mission Hospital, Seboche, in northern Lesotho between 01 August 2021 and 31 July 2022 with symptoms of respiratory infection (cough, shortness of breath or sore throat). As part of the parent study procedures, all participants underwent on‐site SARS‐CoV‐2 rapid diagnostic testing (RDT), with a subset also receiving on‐site PCR testing. Aliquots of the nasopharyngeal swab samples used for RDT were stored at −80 °C for subsequent multiplex PCR testing for 18 viruses and 4 bacteria, including SARS‐CoV‐2 (BIOFIRE RP 2.1 plus).

**Results:**

Of the 511 samples tested, 161 (31.5%) were positive for one pathogen and five (1.0%) for two pathogens. The most common pathogens were SARS‐CoV‐2 (41.6%), human rhinovirus/enterovirus (36.7%), non–COVID human coronaviruses (6.6%), parainfluenza viruses (6%) and influenza A and B viruses (4.8%). Human rhinoviruses/enteroviruses and SARS‐CoV‐2 showed a counter‐cyclical pattern. Seasonal patterns were observed for human rhinoviruses/enteroviruses, human coronaviruses, parainfluenza and influenza A and B viruses.

**Conclusion:**

In this study, viral upper respiratory infections in Lesotho showed a pathogen spectrum and seasonal patterns similar to those described in other temperate climate settings.

## 1. Introduction

Viral and bacterial respiratory tract infections are associated with high morbidity and mortality worldwide. In 2021, upper respiratory tract infections (URTIs) alone accounted globally for 12.8 billion episodes of illness and 19,600 deaths with the highest mortality rates seen in Africa [1].

Although URTIs cause fewer deaths and disability when compared to lower respiratory tract infections (LRTIs), they constitute a significant burden for the healthcare systems and society at large, due to higher medical expenses, workplace absenteeism and lower work productivity overall [1, 2]. Moreover, URTIs can progress into LRTIs with high morbidity and mortality [1, 3]. Possible pathogens causing URTIs include influenza viruses, parainfluenza viruses, respiratory syncytial virus (RSV), rhinoviruses, coronaviruses, *Streptococcus pneumoniae*, *Haemophilus influenza* and *Mycoplasma pneumoniae* [1].

The seasonal patterns of the most common viral pathogens of respiratory tract infections are well documented in most regions with temperate climate zones [4, 5]. However, data on URTIs and their seasonality in Africa remain comparatively scarce and less conclusive. Studies performed in tropical and subtropical countries in Africa showed overall comparable viral spectra, following less consistent and pronounced viral seasonality, largely in line with the dry and wet seasons, sometimes varying across different regions within a single country [6–10]. In contrast, studies from South Africa showed viral seasonality following the known patterns from other temperate climate zones but with overall wider and flatter seasonal peaks [6, 11, 12]. Possible reasons for the observed differences in viral seasonality may include the effects of less pronounced seasonal variations in terms of temperature and humidity, ecological and sociodemographic differences and differing rates of comorbidities, such as HIV infection [13].

Lesotho, a lower middle‐income country surrounded by South Africa, faces a triple burden of disease, characterised by some of the highest rates of HIV and tuberculosis worldwide, high morbidity and mortality due to injuries, alongside a rising prevalence of noncommunicable diseases (NCDs) [14–18]. A literature search revealed no epidemiological data on causes of respiratory infections other than tuberculosis or severe acute respiratory syndrome coronavirus 2 (SARS‐CoV‐2) from Lesotho [19]. Though recent data from South Africa are available, the unique context of Lesotho, with its predominantly rural population, mountainous highlands, temperate to subtropical climate and higher rates of HIV and tuberculosis, may influence the pathogen spectrum in this setting [14].

The aim of this study was to describe the spectrum of pathogens causing respiratory infections in adults in Lesotho over the course of one year from 1 August 2021 to 31 July 2022 and to explore for a potential seasonal pattern.

## 2. Methods

Causes and seasonality of upper respiratory infections in adults in Lesotho (CORIAL) was a prospectively planned cross‐sectional study nested in the MistraL project (Mitigation strategies for communities with COVID‐19 transmission in Lesotho using artificial intelligence on chest X‐rays and novel rapid diagnostic tests) [20]. The MistraL project provided same‐day, one‐stop testing for coronavirus disease 2019 (COVID‐19), HIV and tuberculosis during the peak of the COVID‐19 pandemic in northern Lesotho. The MistraL study’s participants were people with presumptive COVID‐19 who were tested for SARS‐CoV‐2 using one or two nasopharyngeal swabs, one for a rapid diagnostic test (RDT) (STANDARD Q COVID‐19 Antigen test, SD Biosensor, Republic of Korea) and in a subset one for an on‐site polymerase chain reaction (PCR) test (ABI 7500 Real‐Time PCR, Applied Biosystems, USA and Xpert Xpress SARS‐CoV‐2, Cepheid, USA). These tests were performed to validate diagnostic tests in the Southern African context. The detailed design and outcomes of the MistraL project have been reported previously [20, 21]. The CORIAL study utilised an aliquot from the nasopharyngeal sample that was used for the RDT. CORIAL aimed at investigating causes and seasonality of URTIs in adults in Lesotho.

### 2.1. Setting

CORIAL was conducted at St. Charles Mission Hospital, Seboche, a church‐led hospital in Butha‐Buthe district providing primary and secondary care. Butha‐Buthe is a mostly rural, mountainous district in northern Lesotho, bordering South Africa, with an estimated population of about 118,000 [22, 23]. In 2023, Lesotho’s adult HIV prevalence was estimated at 18.5% and the tuberculosis incidence at 664/100,000 [16, 17].

### 2.2. Participants

All MistraL participants aged 18 or older, between 27 January 2021 and 22 August 2022, were screened for participation in CORIAL. We limited participation to adults as MistraL, the parent study of CORIAL, did not include paediatric patients aged 5 years or less. MistraL participants with at least one of the three following predefined symptoms (cough of any duration, shortness of breath or sore throat) and providing written informed consent were eligible.

### 2.3. Study Procedures

All participants had at least one nasopharyngeal SARS‐CoV‐2 RDT performed for the MistraL study. The sample for the analyses of CORIAL was obtained from the remaining volume of the nasopharyngeal Standard Q RDT testing tube. After collection, the tubes were brought to the laboratory of St. Charles Mission Hospital, Seboche, where they were pipetted into microtubes, labelled and frozen at −80°C. The samples were shipped frozen to the Department of Clinical Bacteriology/Mycology at the University Hospital Basel, Switzerland. In Switzerland, the samples were screened for 22 viral and bacterial respiratory pathogens, including SARS‐CoV‐2, using a commercial multiplex PCR BIOFIRE Respiratory Panel 2.1 plus (bioMérieux) according to the protocol of the manufacturer. The pathogens covered by the PCR BIOFIRE are listed in​ Table 1.

**TABLE 1:** Multiplex PCR and SARS‐CoV‐2 testing.

Results	Count (proportion)
Total of individuals tested *N* = 511	511 (100%)
Positive swabs	161 (31.5%)
More than 1 pathogen	5 (1.0%)
Number of pathogens detected *N* = 166	166 (100%)
SARS‐CoV‐2 (RDT and PCR)^$^	69 (41.6%)
Human rhinovirus/enterovirus	61 (36.7%)
Influenza A virus	6 (3.6%)
Influenza A virus A/H1 2009	4 (2.4%)
Influenza A virus, no subtype detected	1 (0.6%)
Influenza A virus, equivocal	1 (0.6%)
Influenza A virus A/H3	0 (0%)
Influenza B virus	2 (1.2%)
Parainfluenza virus 1	3 (1.8%)
Parainfluenza virus 3	1 (0.6%)
Parainfluenza virus 4	6 (3.6%)
Coronavirus HKU1	5 (3.0%)
Coronavirus NL63	2 (1.2%)
Coronavirus OC43	4 (2.4%)
Adenovirus	2 (1.2%)
Respiratory syncytial virus	2 (1.2%)
Human metapneumovirus	1 (0.6%)
*Mycoplasma pneumoniae*	2 (1.2%)
Coronavirus 229E	0 (0%)
Parainfluenza virus 2	0 (0%)
*Bordetella pertussis*	0 (0%)
*Bordetella parapertussis*	0 (0%)
*Chlamydia pneumoniae*	0 (0%)
Coinfections *N* = 511	5 (1.0%)
Human rhinovirus/enterovirus + SARS‐CoV‐2	2 (0.4%)
Human rhinovirus/enterovirus + parainfluenza virus 4	1 (0.2%)
Coronavirus HKU1 + coronavirus OC43	1 (0.2%)
Parainfluenza virus 1 + parainfluenza virus 3	1 (0.2%)

Abbreviations: PCR, polymerase chain reaction; RDT, rapid diagnostic test.

^$^25 cases were identified using on‐site PCR in Lesotho, 54 using on‐site RDT in Lesotho and 11 using multiplex PCR in Switzerland. On‐site PCR and RDT identified 68 cases, and multiplex PCR identified 1 additional case.

### 2.4. Study Participant Sampling

From the samples provided by MistraL study participants eligible for and consenting to CORIAL during the final 12‐month observation period (1 August 2021–31 July 2022), we randomly selected samples from 511 participants using a random number generator in RStudio (R 4.3.1). Our sampling aimed for an even distribution of participants across all 12 months, targeting 43 participants per month.

### 2.5. Data Analysis

All analyses were run in RStudio (R 4.3.1). This was an explorative and descriptive study without a specific hypothesis. There was no predefined sample size. The target sample size of 511 analyses by multiplex PCR was based on available funding.

Baseline clinical data and test results for HIV, tuberculosis and SARS‐CoV‐2 were taken from the MistraL project database and merged with the multiplex PCR test results from the laboratory of the Department of Clinical Bacteriology/Mycology at University Hospital Basel, Switzerland. A COVID‐19 case was defined as a positive PCR result—either from the multiplex PCR in Switzerland or the on‐site MistraL PCR in Lesotho—or at least one positive on‐site SARS‐CoV‐2 RDT result. We opted for this definition as not all participants received all three test modalities.

### 2.6. Ethical Approval

CORIAL was a substudy of the MistraL study protocol that was approved by the National Health Research and Ethics Committee (NH‐REC) of Lesotho (ID 107‐2020) and by the Ethics Committee Switzerland (Ethikkommission Nordwest‐ und Zentralschweiz (EKNZ) AO_2020‐00018). The MistraL consent form included the consent for shipment of nasopharyngeal samples to the laboratory of the Department of Clinical Bacteriology/Mycology at University Hospital Basel, Switzerland, and its analysis through multiplex PCR. All MistraL participants provided signed informed consent. Persons who could not write provided consent with a thumbprint, and a witness not involved in the study signed on their behalf. All procedures were conducted in accordance with the approved protocol and ethical standards from the Declaration of Helsinki [24].

## 3. Results

**FIGURE 1:**
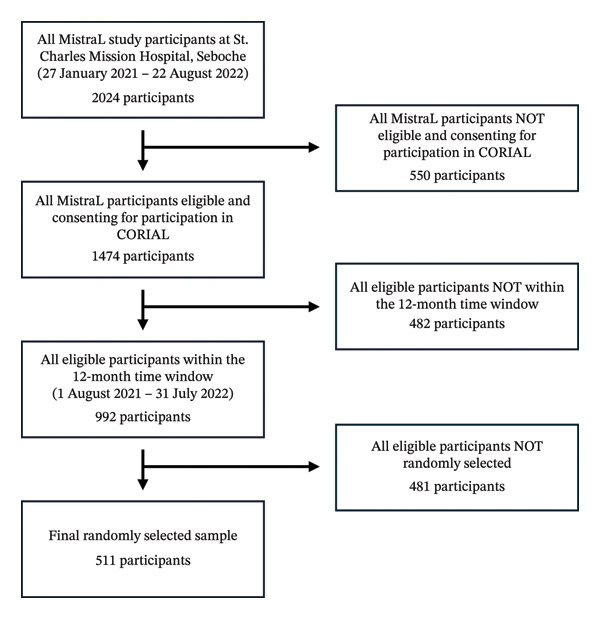
Participant flowchart.

**FIGURE 2:**
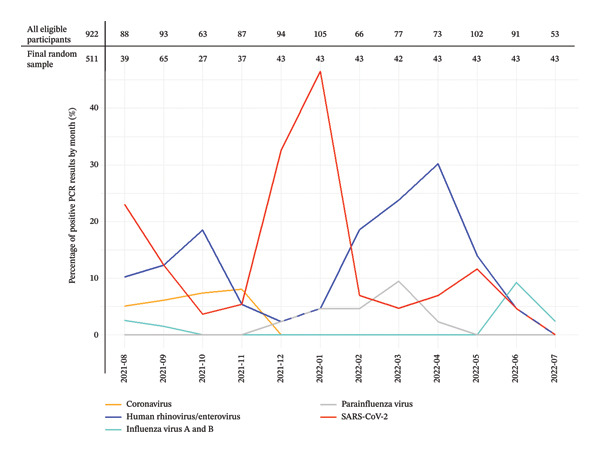
Monthly URTI pathogen rates in the study sample from August 2021 to July 2022.

Out of the total 2024 participants from the MistraL study in St. Charles Mission Hospital, Seboche (27 January 2021–22 August 2022), 1474 (72.8%) were eligible for participation in CORIAL according to the clinical eligibility criteria and consented. Among them, 992 (49.0%) participants were within our defined 12‐month observation window (1 August 2021–31 July 2022). After the random selection of samples to be analysed using the multiplex PCR, 511 (25.2%) participants were included in the analysis (Figure 1). The median number of participants included per month was 43, the lowest being 27 (October 2021) and the highest being 65 participants (September 2021) (Figure 2).

Table 2 provides an overview of the baseline characteristics of the CORIAL study population. The most common reported symptoms were cough (428, 83.8%), sore throat (133, 26.0%) and shortness of breath (118, 23.1%). Overall, 292 were female (57.1%) and the median age was 47 years (interquartile range 33–63), 116 (22.7%) were 65 years or older. Overweight (BMI ≥ 25 kg/m^2^) was recorded in 259/507 (51.1%), 100 (19.6%) reported having high blood pressure, 42 (8.2%) reported to smoke and 55 (10.8%) reported having ever worked in mines. Overall, 119 participants (23.3%) had HIV, of whom 110 (92.4%) were taking ART. Information on the degree of immunosuppression was not available. A total of 37 participants (7.2%) had tuberculosis.

**TABLE 2:** Participants’ characteristics and self‐reported symptoms.

Baseline characteristics	Count (proportion)
Total	511 (100%)
Gender	
Female	292 (57.1%)
Male	219 (42.9%)
Age	
Median (IQR)	47 (33–63)
≥ 65 years	116 (22.7%)
BMI N = 507*^∗^*	
Median (IQR)	25.3 (21.4–30.2)
Underweight BMI < 18.5 kg/m^2^	47 (9.3%)
Normal weight BMI 18.5–24.9 kg/m^2^	201 (39.6%)
Overweight BMI 25–29.9 kg/m^2^	127 (25.0%)
Obesity (Class I–III) BMI ≥ 30 kg/m^2^	132 (26.0%)
HIV status	
People with HIV taking ART	110 (21.5%)
People with HIV not taking ART	4 (0.8%)
People newly diagnosed with HIV	5 (1.0%)
People without HIV, last test < 12 months	390 (76.3%)
People without HIV, last test ≥ 12 months	2 (0.4%)
Tuberculosis status	
People with TB and an ongoing treatment	9 (1.8%)
People newly diagnosed with TB, GeneXpert positive	9 (1.8%)
People newly diagnosed with TB, clinical diagnosis	19 (3.7%)
People without TB	474 (92.8%)
Other comorbidities and risk factors (self‐reported)	
High blood pressure	100 (19.6%)
Diabetes	39 (7.6%)
Currently smoking	42 (8.2%)
< 10 cigarettes/day	35 (6.8%)
≥ 10 cigarettes/day	7 (1.4%)
Alcohol consumption (≥ 2 times/week)	19 (3.7%)
Ever worked in a mine	55 (10.8%)
Symptoms (self‐reported) *N* = 511	Count (proportion)
Cough	428 (83.8%)
Duration in days (median, IQR), *N = 428*	5 (3–8)
Producing sputum, *N = 428*	268 (62.6%)
Coughing blood, *N = 428*	8 (1.9%)
Sore throat	133 (26.0%)
Duration in days (median, IQR) *N = 133*	4 (2–6)
Shortness of breath	118 (23.1%)
Duration in days (median, IQR) *N = 118*	5 (3–7)
Fever (≥ 38°C)	0 (0%)
Fatigue	93 (18.2%)
Chest pain	65 (12.7%)
Headache	46 (9.0%)
Muscle pain	36 (7.0%)
Night sweat	22 (4.3%)
Weight loss	16 (3.1%)
Diarrhoea	12 (2.3%)
Running nose	12 (2.3%)
Loss of sense of smell or taste	8 (1.6%)
Vomiting	5 (1.0%)
Skin rash	2 (0.4%)

Abbreviations: ART, antiretroviral therapy; BMI, body mass index; IQR, interquartile range; TB, tuberculosis.

^∗^ = 100%, missing data in 4 participants.

### 3.1. Outcomes of Multiplex PCR and SARS‐CoV‐2 Testing

Table 1 shows the results of the multiplex PCR and the SARS‐CoV‐2 testing. Out of the 511 tested samples, 161 (31.5%) were positive for at least one pathogen. Five (1.0%) were positive for more than one pathogen. In total, 166 pathogens were detected. The most common pathogen was SARS‐CoV‐2 (41.6%), followed by human rhinovirus/enterovirus (36.7%) and the groups of non–COVID human coronaviruses (6.6%), parainfluenza viruses (6%) and influenza A and B viruses (4.8%). Only few instances of adenovirus, RSV and human metapneumovirus were detected.

We observed a total of five coinfections using the multiplex PCR, three of them involving human rhinovirus/enterovirus (Table 1). Coinfections with HIV and tuberculosis are described in Supporting Table 1. Of the 119 people with HIV, 37 (31.1%) samples were positive for URTI viral pathogens. Of the 37 people with tuberculosis, 4 (10.8%) samples were positive for URTI viral pathogens. For both HIV and tuberculosis, the most commonly detected pathogens were human rhinovirus/enterovirus and SARS‐CoV‐2. The rates of URTI viral pathogen detection appeared to be similar between people with and without HIV (Supporting Table 2).

### 3.2. Virus Seasonality

The monthly rates of URTI pathogens within the study sample over the 12‐month assessment period are shown in Figure 2. We observed two peaks of SARS‐CoV‐2 infections: from August to September 2021 as well as from December 2021 to January 2022, which coincide with the known COVID‐19 waves in Lesotho [20]. Human rhinovirus/enterovirus was detected year‐round, with two peaks: in the spring, from August to October 2021 and in late summer and autumn, from February to May 2022. Additionally, we observed that the peak of SARS‐CoV‐2 (from December 2021 to January 2022) coincided with a dip in human rhinovirus/enterovirus infections, while the peak of human rhinovirus/enterovirus (from February 2022 to May 2022) coincided with a dip in SARS‐CoV‐2 infections. The influenza viruses (A and B) showed peaks in the winter months (August 2021 and June 2022). Parainfluenza viruses (1, 3 and 4) were observed in the summer and early autumn months, with a peak in March 2022. Coronaviruses (HKU1, NL63 and OC43) were seen in the spring and early summer months (from August to November 2021). The absolute numbers for all pathogens by month are shown in Supporting Table 3.

## 4. Discussion

Among 511 adults presenting with at least one symptom of URTI, between August 2021 and July 2022, we identified in 31.5% a pathogen through nasopharyngeal swabs. The most common pathogens identified were SARS‐CoV‐2, human rhinovirus/enterovirus, non–COVID human coronaviruses, parainfluenza and influenza viruses. We observed a counter‐cyclical pattern of occurrence between SARS‐CoV‐2 and rhino‐/enterovirus infections.

The characteristics of our study population align closely with data from previous studies in Lesotho, suggesting it is likely a realistic representation of the country’s adult population. Rates of self‐reported hypertension and diabetes correspond well with prior findings [25]. SARS‐CoV‐2 followed a time pattern in line with known COVID‐19 lockdown periods in Lesotho [20].

Overall rates of pathogens detected align with previous publications from other settings, including the global burden of disease studies [1, 11, 13, 26]. A recent study from South Africa, which surrounds Lesotho, conducted from April 2020 to March 2021, reports similar findings with some notable differences [11]. In both the South African and our studies, human rhinovirus emerged as the most common pathogen after SARS‐CoV‐2, with detectable year‐round transmission. However, in the South African study, the peaks occurred more in the winter, as opposed to spring and autumn in our study. Human coronaviruses and parainfluenza virus were also detected at similar rates in both studies. In contrast, our study recorded significantly lower rates of RSV and adenovirus. The lower rate of RSV in our study may be explained by an exclusively adult study population where RSV is less often detected compared to children [27].

Our findings on seasonality of the identified viral pathogens are similar to patterns reported from other temperate climate settings [4, 5]. Human rhinovirus/enterovirus was detectable all year with peaks in spring (from August to October 2021) and late summer/autumn (from February to May 2022), while influenza viruses showed peaks in the winter months (August 2021 and June 2022). Parainfluenza viruses were observed in the summer months, peaking in early autumn (March 2022), especially parainfluenza virus 1. Our results for human coronaviruses deviated slightly from the literature, being detectable all spring, as opposed to previously described peaks in winter and early spring [4, 5].

We observed a counter‐cyclical pattern between SARS‐CoV‐2 and human rhinovirus/enterovirus infections, and few coinfections. Previous literature suggests that variations in non–SARS‐CoV‐2 virus epidemiology may depend on antagonistic competition between SARS‐CoV‐2 and other respiratory viruses. For instance, it has been shown that subjects with influenza have a lower risk of COVID‐19 and that a similar correlation can be demonstrated for COVID‐19 and rhinoviruses. However, the degree of interference is virus‐specific, and in the case of rhino‐ and enterovirus, the literature suggests a stronger correlation with lockdown and social distancing measures, with prevalences rebounding and persisting after easement of lockdown measures [28–31]. Another explanation for this observed counter‐cyclicity might be the sampling effect that we possibly introduced by aiming to randomly sample an equal number of participants across all 12 months. Since the overall number of patients between months fluctuated, especially during SARS‐CoV‐2 waves, sampling a hypothetically stable level of human rhinovirus/enterovirus population during such a wave could show up as dip in the relative plot.

The strengths of this study include its efficient nested study design, the inclusion of a broad adult population presenting with URTI symptoms—largely representative of Lesotho’s general population—and the availability of full‐year data, allowing for the assessment of seasonality [25].

The study has, however, several limitations. Firstly, even though URTIs are known to have the highest burden in paediatric, particularly neonatal age groups, our study focused exclusively on adults. The original recruitment for the MistraL study did not allow for the inclusion of paediatric patients aged 5 years or younger. For this reason, we decided to focus exclusively on the adult population [20].

Another limitation of this nested study is the use of different diagnostic standards for SARS‐CoV‐2 and other respiratory pathogens. SARS‐CoV‐2 cases were defined as any positive result on PCR or RDT, reflecting the parent study MistraL’s primary focus on COVID‐19, which followed the same approach. All other respiratory viruses were identified exclusively by multiplex PCR (RP 2.1 plus). This may have created differences in diagnostic sensitivity and specificity, potentially inflating SARS‐CoV‐2 detection rates. However, since this study primarily focused on non–SARS‐CoV‐2 pathogens, which were assessed using a consistent diagnostic approach, SARS‐CoV‐2 results mainly served to contextualise findings for the broader spectrum of upper respiratory pathogens.

Other limitations of this study include a comparatively low sample size and positivity rate of multiplex PCR samples. Additionally, because data on immunosuppression levels were unavailable, we could not examine associations between CD4 counts and URTI pathogens. Lastly, this study’s generalisability to other settings and time periods in Lesotho may be limited due to the single‐centre design and the timing of the data collection, which overlapped with SARS‐CoV‐2 waves and related lockdowns that may have influenced transmission patterns of the other respiratory pathogens.

### 4.1. Implications

This observational study provides a first insight into the pathogen spectrum and seasonality of viral URTI in adults in northern Lesotho. Our results suggest that URTIs in Lesotho generally have a pathogen spectrum and seasonal patterns similar to those in other areas of the world with a temperate climate. However, because this study overlapped with SARS‐CoV‐2 waves, its generalisability to other periods may be limited, highlighting the need for further postpandemic research.

## Funding

This work was supported by Botnar Research Centre for Child Health (BRCCH) as part of the Multi‐Investigator Project/Fast Track Call for Acute Global Health Challenges (grant numbers: DZX2167 and DZX2168). N.D.L. received his salary through a SNSF Eccellenza Professorship grant​ (PCEFP3‐181355). Open‐access publishing facilitated by Universitat Basel, as part of the Wiley–Universitat Basel agreement via the Consortium Of Swiss Academic Libraries.

## Conflicts of Interest

The authors declare no conflicts of interest.

## Supporting Information

Supporting Table 1 visualises coinfection of the detected URTI pathogens in people living with HIV and tuberculosis.

Supporting Table 2 visualises the rates of URTI pathogen detection in people with and without HIV.

Supporting Table 3 shows the absolute numbers of detected URTI pathogens for each pathogen by month.

## Supporting information


**Supporting Information** Additional supporting information can be found online in the Supporting Information section.

## Data Availability

The data that support the findings of this study are openly available in Zenodo at https://zenodo.org/, reference number 10.5281/zenodo.18375714.
