# Supplementary material for: Causes and Seasonality of Upper Respiratory Infections in Adults in Lesotho (2021–2022) (CORIAL)
Source: Can J Infect Dis Med Microbiol. 2026 Feb 19;2026:3732614. doi: 10.1155/cjid/3732614 (PMC12920662; doi:10.1155/cjid/3732614)
Supplement: Supplementary file 1 — Supporting Information Additional supporting information can be found online in the Supporting Information section. [file CJID-2026-3732614-s001.docx]

**Supplement**

| **Co-infections HIV and tuberculosis** | Count (Proportion) |
| --- | --- |
|  |  |
| **Total of individuals tested** N = 511 | 511 (100%) |
|  |  |
| **People with HIV and tuberculosis** N = 511 | 14 (2.7%) |
| Negative swabs | 13 (2.5%) |
| Human rhinovirus/enterovirus | 1 (0.2%) |
|  |  |
| **People with HIV** N = 511 | 119 (23.3%) |
| Positive swabs | 37 (7.2%) |
| Human rhinovirus/enterovirus | 15 (2.9%) |
| SARS-CoV-2 | 14 (2.7%) |
| Parainfluenza virus 4 | 2 (0.4%) |
| Coronavirus HKU1 | 1 (0.2%) |
| Coronavirus OC43 | 1 (0.2%) |
| Influenza A (no subtype detected) | 1 (0.2%) |
| Influenza A H1-2009 | 1 (0.2%) |
| Influenza B | 1 (0.2%) |
| Parainfluenza virus 1 and parainfluenza virus 3 | 1 (0.2%) |
|  |  |
| **People with tuberculosis** N = 511 | 37 (7.2%) |
| Positive swabs | 4 (0.8%) |
| Human rhinovirus/enterovirus | 2 (0.4%) |
| SARS-CoV-2 | 1 (0.2%) |
| Influenza A equivocal | 1 (0.2%) |

***Table 1: Co-infections with HIV and tuberculosis***

***Table 2: Comparison of pathogen detection rates between people living with and without HIV***

| **HIV** | **Participants (Proportion)** | **Positive swabs** | **SARS-CoV-2** | **Human rhinovirus / enterovirus** | **Tuberculosis** |
| --- | --- | --- | --- | --- | --- |
|  |  |  |  |  |  |
| **Total**  N = 511 | 511 (100%) | 161  (31.5%) | 69  (13.5%) | 61  (11.9%) | 37  (7.2%) |
| **People with HIV** N = 119 | 119  (23.3% of 511) | 37  (31.1%) | 14  (11.8%) | 15  (12.6%) | 14  (11.8%) |
| **People without HIV** N = 392 | 392 (76.7% of 511) | 124  (31.6%) | 55  (14.0%) | 46  (11.7%) | 23  (5.9%) |

***Table 3: Absolute numbers of all detected pathogens for each month.***

|  | Total amount of pathogens detected (N = 166) | Aug 2021 | Sep 2021 | Oct 2021 | Nov 2021 | Dec 2021 | Jan 2022 | Feb 2022 | Mar 2022 | Apr 2022 | May 2022 | Jun 2022 | Jul 2022 |
| --- | --- | --- | --- | --- | --- | --- | --- | --- | --- | --- | --- | --- | --- |
| **Total amount of samples taken per month (**N = 511) |  | 39 | 65 | 27 | 37 | 43 | 43 | 43 | 42 | 43 | 43 | 43 | 43 |
| **Total amount of positive samples per month (N = 161)** |  | 19 | 20 | 8 | 7 | 16 | 25 | 13 | 13 | 17 | 11 | 10 | 2 |
| **Total amount of pathogens per month** | 166 (100%) | 19 | 21 | 8 | 7 | 16 | 25 | 13 | 15 | 19 | 11 | 10 | 2 |
| SARS-CoV-2 | 69 (41.6%) | 9 | 8 | 1 | 2 | 14 | 20 | 3 | 1 | 4 | 5 | 2 |  |
| Human rhinovirus/enterovirus | 61 (36.7%) | 4 | 8 | 5 | 2 | 1 | 2 | 8 | 10 | 13 | 6 | 2 |  |
| Influenza A H1-2009 | 4 (2.4%) |  |  |  |  |  |  |  |  |  |  | 3 | 1 |
| Influenza A (no subtype) | 1 (0.6%) |  |  |  |  |  |  |  |  |  |  | 1 |  |
| Influenza A equivocal | 1 (0.6%) |  |  |  |  |  |  |  |  |  |  | 1 |  |
| Influenza B | 2 (1.2%) | 1 | 1 |  |  |  |  |  |  |  |  |  |  |
| Parainfluenza virus 1 | 3 (1.8%) |  |  |  |  |  |  | 1 | 2 |  |  |  |  |
| Parainfluenza virus 3 | 1 (0.6%) |  |  |  |  |  |  |  | 1 |  |  |  |  |
| Parainfluenza virus 4 | 6 (3.6%) |  |  |  |  | 1 | 2 | 1 | 1 | 1 |  |  |  |
| Coronavirus HKU1 | 5 (3.0%) | 1 | 2 |  | 2 |  |  |  |  |  |  |  |  |
| Coronavirus NL63 | 2 (1.2%) | 1 |  | 1 |  |  |  |  |  |  |  |  |  |
| Coronavirus OC43 | 4 (2.4%) |  | 2 | 1 | 1 |  |  |  |  |  |  |  |  |
| Adenovirus | 2 (1.2%) | 2 |  |  |  |  |  |  |  |  |  |  |  |
| Respiratory syncytial virus | 2 (1.2%) | 1 |  |  |  |  |  |  |  | 1 |  |  |  |
| Human metapneumovirus | 1 (0.6%) |  |  |  |  |  | 1 |  |  |  |  |  |  |
| Mycoplasma pneumoniae | 2 (1.2%) |  |  |  |  |  |  |  |  |  |  | 1 | 1 |
| Coronavirus 229E | 0 (0%) |  |  |  |  |  |  |  |  |  |  |  |  |
| Influenza A virus A/H3 | 0 (0%) |  |  |  |  |  |  |  |  |  |  |  |  |
| Parainfluenza virus 2 | 0 (0%) |  |  |  |  |  |  |  |  |  |  |  |  |
| Bordetella pertussis | 0 (0%) |  |  |  |  |  |  |  |  |  |  |  |  |
| Bordetella parapertussis | 0 (0%) |  |  |  |  |  |  |  |  |  |  |  |  |
| Chlamydia pneumoniae | 0 (0%) |  |  |  |  |  |  |  |  |  |  |  |  |
